# A Vector-Based Method to Analyze the Topography of Glial Networks

**DOI:** 10.3390/ijms20112821

**Published:** 2019-06-10

**Authors:** Sara Eitelmann, Jan J. Hirtz, Jonathan Stephan

**Affiliations:** 1 Animal Physiology Group, Department of Biology, University of Kaiserslautern, Erwin Schrödinger-Straße 13, D 67663 Kaiserslautern, Germany; sara.eitelmann@bio.uni-kl.de; 2 Physiology of Neuronal Networks Group, Department of Biology, University of Kaiserslautern, Erwin Schrödinger-Straße 13, D 67663 Kaiserslautern, Germany; hirtz@bio.uni-kl.de

**Keywords:** anisotropy, connexin, sulforhodamine 101, astrocyte, oligodendrocyte, gap junctions, tracer, neurobiotin, LSO, lateral superior olive

## Abstract

Anisotropy of tracer-coupled networks is a hallmark in many brain regions. In the past, the topography of these networks was analyzed using various approaches, which focused on different aspects, e.g., position, tracer signal, or direction of coupled cells. Here, we developed a vector-based method to analyze the extent and preferential direction of tracer spreading. As a model region, we chose the lateral superior olive—a nucleus that exhibits specialized network topography. In acute slices, sulforhodamine 101-positive astrocytes were patch-clamped and dialyzed with the GJ-permeable tracer neurobiotin, which was subsequently labeled with avidin alexa fluor 488. A predetermined threshold was used to differentiate between tracer-coupled and tracer-uncoupled cells. Tracer extent was calculated from the vector means of tracer-coupled cells in four 90° sectors. We then computed the preferential direction using a rotating coordinate system and post hoc fitting of these results with a sinusoidal function. The new method allows for an objective analysis of tracer spreading that provides information about shape and orientation of GJ networks. We expect this approach to become a vital tool for the analysis of coupling anisotropy in many brain regions.

## 1. Introduction

Cell–cell coupling among glial cells is mediated by gap junction (GJ) channels consisting of connexins (Cx) [1,2]. Astrocyte–astrocyte (A:A) and oligodendrocyte-oligodendrocyte (O:O) coupling are mediated via Cx43 or Cx30 and Cx47 or Cx32 homotypic GJ channels, respectively [1,3]. In addition, astrocytes and oligodendrocytes form panglial A:O networks [4,5,6,7,8]. It was shown that tracer-coupled networks exhibit a region-dependent heterogeneous topography. Spherical networks are found, e.g., in hippocampus (HC) and parts of the neocortex (Ctx) [9,10], whereas anisotropic networks are present in many other systems, e.g., barrel Ctx [11], barreloid thalamus (Th) [7], trigeminal main sensory nucleus [12,13], as well as the lateral superior olive (LSO) [4] and inferior colliculus (IC) [5] in the auditory brainstem. The coupling anisotropy is linked to the anisotropy of astrocytes—an astrocyte occupying an oval territory gives rise to an oval tracer-coupled network [4,5,14,15]. Furthermore, the anisotropy of astrocytes and tracer-coupled networks in HC depends on the expression of Cx30 [15]. However, we have shown that in LSO and IC—two nuclei of the auditory brainstem—respective anisotropy is independent of Cx30 expression, as Cx30 levels are virtually absent in early postnatal stages when coupling experiments were carried out [4,5].

In the past, four different approaches were used to describe the topography of anisotropic tracer-coupled networks: (1) A simple, fast, and often used approach is to measure the extent of tracer spreading in two directions orthogonal to each other [4,5,10,11,15]. However, density and distribution of coupled cells are not taken into account by this method. (2) In another approach, the term “coupling anisotropy” (*C_A_*) was introduced [14]. There, the product of network extension multiplied with the somatic tracer (fluorescence) signal intensity for two directions orthogonal to each other is calculated. (3) In a third approach, the labeling intensity of somata and processes is analyzed to determine network anisotropy [7]. The intensity plot profiles of two rectangles orthogonal to each other and the ratio of respective full-width at half-maximum (FWHM) is calculated. Here, the position and number of coupled cells is neglected. (4) Finally, a method including automated cell detection was developed [12,13]. After identification of coupled cells, the vector sum is calculated to describe the preferential spreading of the GJ tracer. So far, these different approaches were never applied to the same data set to assess to which extent the results might depend on the analysis strategy.

**Figure 1:**
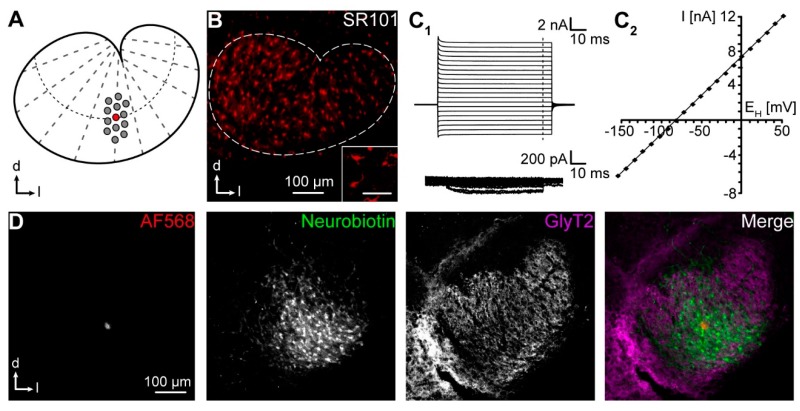
Gap junction networks. (**A**) Scheme depicting the preferential orientation of GJ networks in the lateral superior olive (LSO). (**B**) SR101-labeling of LSO astrocytes. The white dotted line indicates LSO borders. Inset: Astrocytes at higher magnification from the same preparation. Scale bar: 30 µm. (**C**) Electrophysiological properties. An astrocyte was recorded in voltage-clamp mode and stepwise hyper- and depolarized (**C_1_**). Membrane currents were recorded before (top) and after isolation of voltage-dependent currents (*p*/4, bottom). Inward currents reflect weak inward rectification that is typical for mature astrocytes. The *I*/*V* relationship was determined at the end of the voltage steps (dashed line in **C_1_**). The *I*/*V* relationship was linear, which is typical for mature astrocytes (**C_2_**). (**D**) The tracer neurobiotin (GJ-permeable) diffused from a patch-clamped astrocyte (AF568, GJ-impermeable) to neighboring cells (neurobiotin). Immunohistochemical labeling of the glycine transporter (GlyT) 2 was used to generally highlight the morphology of the LSO. Thereby, the position of the network within the LSO can be determined [4]. Experiment ID: 18-04-25_S2.

We here utilize the LSO, a tonotopically organized part of the auditory brainstem (Figure 1A) [16], as a model system for anisotropic GJ coupling [4]. We identified LSO astrocytes using sulforhodamine (SR) 101, which labels astrocytes in various brain regions [12,17,18,19,20]. Topography of tracer-filled networks was analyzed using the aforementioned approaches. Furthermore, we developed a new vector-based method with subsequent meta-analysis to take different aspects of analysis into account that were incompletely covered by former approaches, i.e., anisotropy of networks, preferred direction of tracer spreading, as well as distribution of coupled glia.

## 2. Results

### 2.1. Astrocyte Networks

LSO astrocytes were identified a priori by SR101-labeling (Figure 1B) [4,17], and were subsequently patch-clamped to determine their basic electrophysiological properties. They exhibited membrane properties typical for astrocytes, i.e., a negative membrane potential (−82.9 ± 0.5 mV, *n* = 63/51) and a low membrane resistance (3.7 ± 0.6 MΩ, *n* = 63/51) [4,17]. Further, astrocytes were categorized in accordance with their *I*/*V* relationship. Cells that primarily expressed ohmic currents (Figure 1C_1_) and hence displayed a preferentially linear *I*/*V* relationship with weak inward rectification [18] (Regression coefficient (*R*^2^) ≥ 0.9983; 68%; Figure 1C_2_) represent passive astrocytes (PA) and cells mainly showing outward currents resulting in a nonlinear *I*/*V* relationship (*R*^2^ < 0.9983; 32%; not shown) represent nonpassive astrocytes (nPA) [5,18]. This is in line with earlier studies showing that LSO astrocytes undergo a developmental transition from nPA towards PA [4,17].

GJ coupling was assessed by simultaneous injection of GJ-permeable tracer neurobiotin into single astrocytes in the central part of the LSO. Afterwards, tracer spread was visualized by labeling with avidin alexa fluor (AF) 488. LSO astrocytes were coupled to dozens of cells (Figure 1D) [4]. Tracer signal was highest in the patched cell and declined with increasing distance.

**Figure 2:**
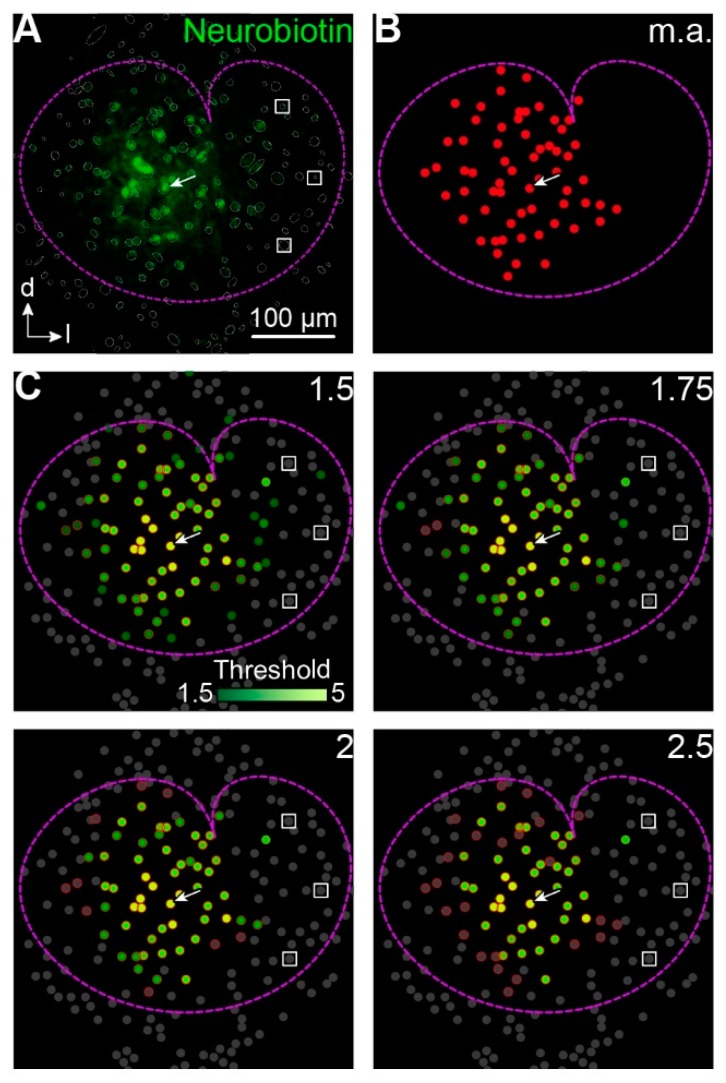

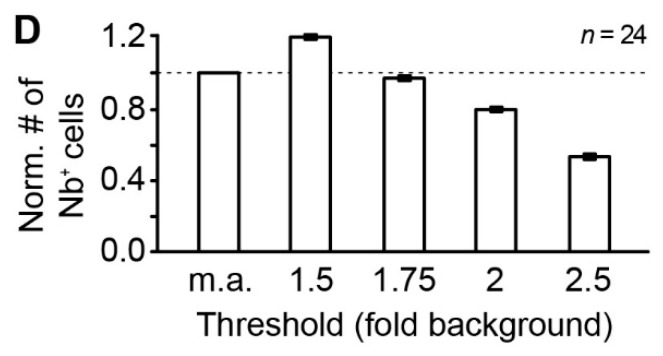
Identification of tracer-coupled cells. (**A**,**B**) Representative tracer-filled network in the center of the LSO (**A**) and schematized representation of manually analyzed (m.a.) coupled cells (**B**). The patched cell (white arrow in **A**–**C**) was identified via labeling with AF568 (not shown; Figure 1D). LSO borders (dotted magenta line) were determined from immunohistochemical labeling of GlyT2 (not shown; Figure 1D). Identified somata in the field of view were encircled (gray circles and ellipses in *A_1_*) for subsequent gray value analysis (**C**). Within LSO borders, three cells were chosen for determination of mean background labeling (white boxes in **A**,**C**) that were distant from the patched cell. (**C**) Dots in C were color-coded depending on the fluorescence intensity of respective cells in A (see scale bar). Cells with a fluorescence intensity of less than the assigned threshold are depicted in gray. Cells that were assigned to the network by manual analysis (**B**) are encircled in red. Experiment ID: 18-04-25_S1 (**A**–**C**). (**D**) The number (#) of neurobiotin-positive (Nb^+^) cells at different thresholds (x-fold background) normalized to values from manually analyzed (m.a.) tracer-filled networks.

Astrocytes and neurons express endogenously biotin [21,22] that can be detected by avidin [23]. Accordingly, it is difficult to judge whether avidin signals in cells in the periphery of the tracer-coupled network result from tracer loading or just from background labeling of endogenous biotin. To overcome the problem of subjectivity in selecting coupled cells we implemented a routine that compares the mean tracer signal of an identified cell with the mean background signal of cells that were located at the edge of the LSO and thus were most likely not tracer-coupled to the patched cell (Figure 2A,C). We chose a threshold of 1.75 times background labeling for further analysis of tracer-coupled networks as here the number of coupled cells matched those values obtained from manual analysis (Figure 2B,D).

### 2.2. Analysis of Network Topography

**Figure 3:**
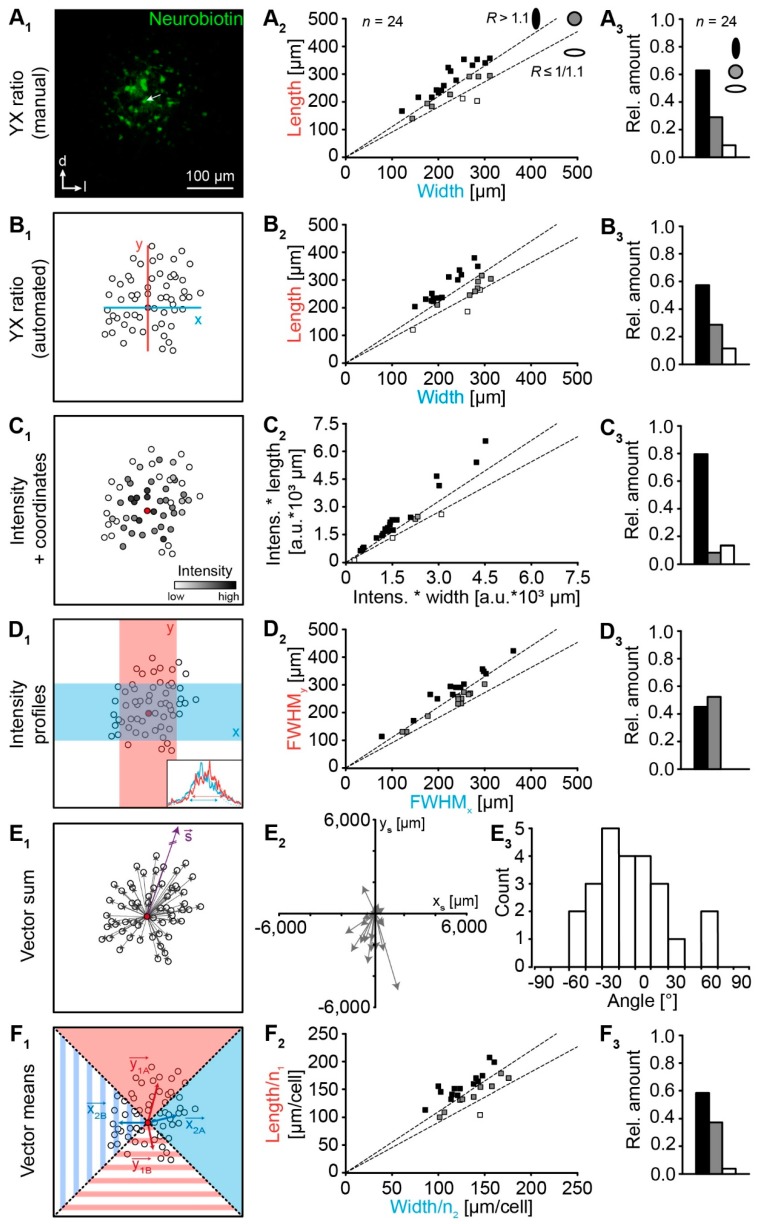
Strategies to analyze gap junction coupling. (**A**) Manually analyzed extension of tracer-filled networks in two directions, which are orthogonal to each other [4,5,10,15]. (**B**) Modification of the strategy shown in **A_1_**. Here, coordinates of tracer-coupled cells that surpass the threshold of 1.75-fold background intensity levels were taken to calculate network extension. (**C**) Preferential tracer spreading was calculated from the product of coordinates and fluorescence intensity of each tracer-coupled cell [14]. (**D**) The histogram of tracer signal in two directions was used to calculate respective full-width at half-maximum (FWHM, inset) [7]. (**E**) The vector sum of all coupled cells was taken to determine the preferential direction of tracer spreading [12,13]. (**F**) The tracer extension was calculated from mean vectors of four 90° sectors. **A_2_–D_2_** and **F_2_** show the determined network extensions in two directions for each experiment by respective strategies of analysis (**A_1_–D_1_,F_1_**). **A_3_–D_3_** and **F_3_** show the affiliation of the tracer-coupled networks to three classes (see Section 4.6). In the majority of approaches, most networks were found to exhibit an oval shape orthogonal to the tonotopic axis. In the vector sum approach, results were heterogeneous and no preferential network orientation was found (**E_2_**_,**3**_). Experiment ID (**A–F**): 18-04-25_S1. The patched cell is marked with a white arrow (**A_1_**) or a filled red symbol (**B_1_–F_1_**).

We next analyzed the topography of tracer-coupled LSO networks using different approaches (Figure 3, Supplementary Table S1). At first, we manually determined the “YX ratio” of tracer spread [4,5,10,11,15]. The majority of LSO networks exhibited an oval shape being oriented orthogonal to the tonotopic axis (class 1; see Section 4.6.), whereas less were spherical (class 2) or oval with an orientation along the tonotopic axis (class 3; Figure 3A). Thus, our new data reproduced the results of our earlier study [4]. We then reanalyzed the topography using further approaches (Figure 3B–F). The results from the automated “YX ratio” approach were much alike, showing a similar distribution of different network classes (Figure 3B). The “Intensity + coordinates” approach [14] gave comparable results, although more tracer-coupled networks were affiliated to class 1 (Figure 3C). In contrast, the “Intensity profiles” approach results only roughly in a comparable distribution (Figure 3D) [7]. Here, tracer-coupled networks were affiliated homogeneously to classes 1 and 2. The recently described “Vector sum” approach [12,13] gave no conclusive results regarding network topography as the direction of main tracer diffusion was highly variable (Figure 3E). Accordingly, this approach was omitted from all further analyses. Finally, the newly developed “Vector means” approach similarly affiliated the networks like the two “YX ratio” approaches before (Figure 3F). It should be mentioned that the network affiliation is largely threshold-independent and only at a threshold of 1.5x background the network affiliation slightly shifts towards class 2 (not shown). However, most of the weakly labeled cells (between 1.5× and 1.75× background) were considered being not efficiently coupled in the manual “YX ratio” approach (Figure 2C). Therefore, this threshold would be chosen to small.

As nPA and PA exhibit different membrane and network properties [17,18,24,25], and we analyzed whether topography and size of LSO networks depended on the maturation state of the astrocyte. Interestingly, the distribution of classes is linked to maturation state of the tracer-loaded astrocyte. nPA and PA preferably gave rise to networks affiliated to class 1 and class 2, respectively (*p* < 0.001; Table 1). Reanalysis of data from previous studies on LSO and IC networks showed similar results (Table 1) [4,5]. Despite these differences, the network size did not depend on the maturation state of astrocytes (nPA: 75 ± 9 cells, *n* = 14; PA: 63 ± 8 cells, *n* = 10; *p* = 0.137).

### 2.3. Meta-Analysis of “Vector Means”

**Figure 4:**
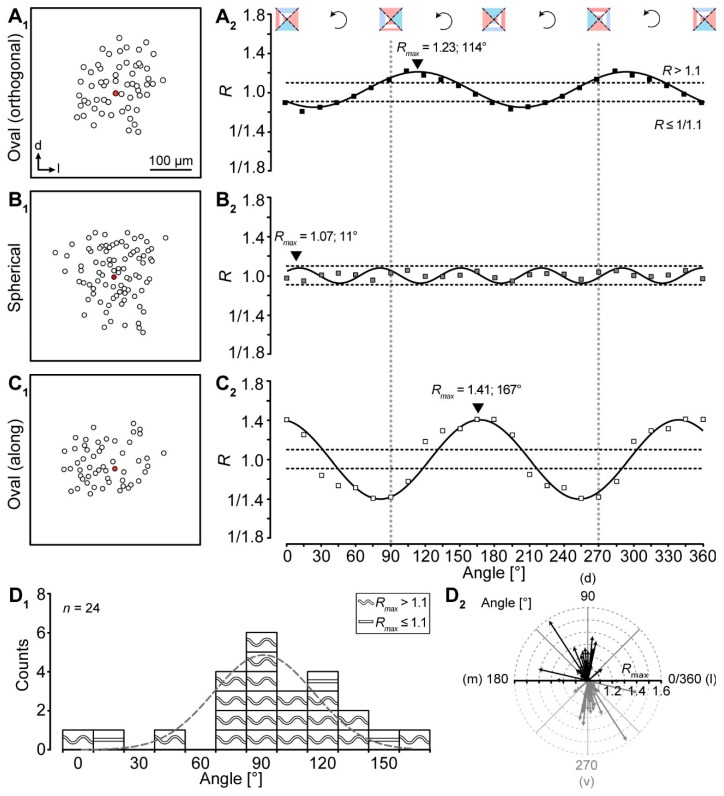
Meta-analysis of gap junction coupling. (**A**–**C**) Examples for differently shaped LSO networks (**A_1_**–**C_1_**). Tracer-coupled networks were analyzed by the “Vector means” approach - the patched cell is marked by a red filled symbol. The preferential direction of tracer spreading was determined using a rotating coordinate system and post hoc fitting of these results with a sinusoidal function (**A_2_**–**C_2_**). The dotted horizontal lines in *A_2_*-*C_2_* represent thresholds for networks to be oval (see Section 4.6). The dotted vertical lines indicate dorsoventral axis that simultaneously reflects the orthogonal to the tonotopic axis. Regression coefficients (*R*^2^): 0.954 (*A_2_*), 0.919 (*B_2_*), and 0.911 (**C_2_**). Experiment IDs: 18-04-25_S1 (**A_1_**), 18-09-05_S3 (**B_1_**), and 18-09-06_S4 (**C_1_**). (**D**) Summary of results showing that most tracer-coupled networks in the central part of the LSO had a preferential dorsoventral (d–v) orientation (**D_1_**_,**2**_) as demonstrated by Gaussian fit (**D_1_**, dotted line) and arrows (**D_2_**). The wave symbol describes an anisotropic, oval topography (*R_max_* > 1.1); the straight line an isotropic, spherical topography (*R_max_* ≤ 1.1).

All ratio-based approaches—including our “Vector means” approach—so far only determine differences of tracer extent in two directions. The topography of a given tracer-coupled network might be concluded wrong by these approaches, e.g., a network might exhibit an oval shape that is rotated by 45° compared to the two directions for which the tracer extent is determined. For such a network, it would be falsely concluded that it might be spherical although it is oval and oriented in a certain direction. To overcome this problem we added a meta-analysis to our “Vector means” approach. We reanalyzed the topography of the tracer-coupled networks using a rotating coordinate system (Figure 4). Thereby, the ratio oscillates two times per full turn in case of anisotropic tracer-coupled networks (classes 1 and 3; Figure 4A_2_,C_2_). In contrast, spherical networks (class 2) show a variable number of oscillations (Figure 4B_2_). A sinusoidal fit was used to determine the angle of maximal anisotropy, i.e., the maximum *R*-value of the fit (*R*_max_). Most networks were anisotropic (21/24; Figure 4D_1_). Furthermore, the majority of these anisotropic networks is oriented roughly orthogonal to the tonotopic axis (16/21; Figure 4D_2_). The remaining 5/21 anisotropic networks were either oriented along the tonotopic axis or “diagonal”. Thus, with the meta-analysis we could confirm the data of our “Vector means” approach (Figure 3F_2,3_): most tracer-coupled networks in the LSO are anisotropic and oriented predominantly orthogonal to the tonotopic axis.

### 2.4. Performance of Approaches

**Figure 5:**
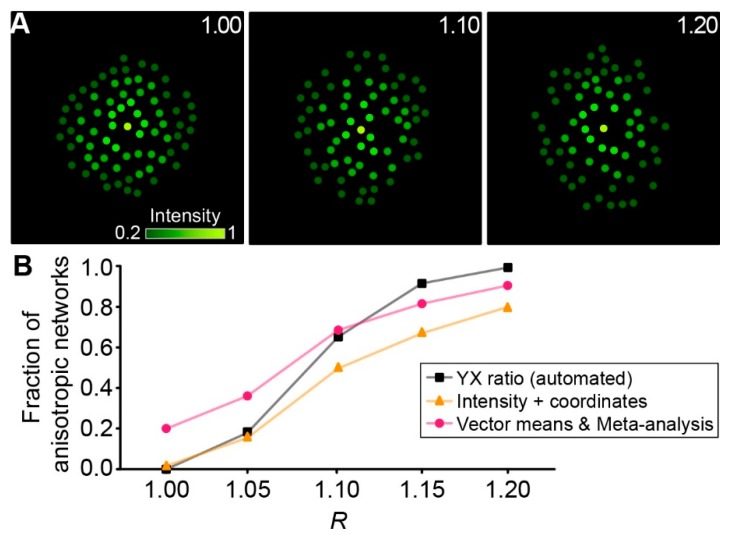
Analysis of in silico networks. (**A**) Artificially generated networks with *R* = 1.00 (left), 1.10 (middle), and 1.20 (right). Dots are color-coded corresponding to the fluorescence intensity of each cell (see scale bar). (**B**) Fraction of anisotropic networks determined with different approaches. For each *R*-value, 50 artificial networks were generated.

Next, we generated artificial networks in silico (see Section 4.6.8.) with a given ratio of length and width to test the capability of different approaches to detect network anisotropy (Figure 5A). Focusing on automated approaches, only automated “YX ratio”, “Intensity + coordinates”, and “Vector means” with subsequent Meta-analysis were compared. The automated “YX ratio” approach performed slightly better than the “Vector means” approach with subsequent meta-analysis, finding more networks to be anisotropic at elevated *R*-values and isotropic at low *R*-values (Figure 5B). In comparison, the “Intensity + coordinates” approach performed relatively poor across the range tested and was not able to compete with the other two approaches.

Taken together, our results show that (1) all ratio-based approaches give similar results, (2) the “Vector sum” approach is not suitable for networks that are symmetric with respect to a point, (3) the meta-analysis of the “Vector means” approach can be used to further characterize topography and orientation of tracer-coupled networks, and (4) our newly developed “Vector means” approach with subsequent meta-analysis and the automated “YX ratio” approach exhibit a high capability to detect network anisotropy.

## 3. Discussion

In the present study, we developed a new vector-based method (“Vector means”), with subsequent meta-analysis for the investigation of gap junctional tracer coupling. Our data demonstrate that our new method outperforms most of the previously described approaches. All ratio-based approaches accurately show an overall similar distribution of differently shaped tracer-coupled networks in the LSO. In addition, our meta-analysis allows a more sophisticated investigation of preferential tracer spread independent from predefined assumptions.

### 3.1. Intensity-Based Cell Detection Method

The unambiguous identification of tracer-filled cells represents a difficult task. The tracer signal exponentially declines from the center to the edge of the tracer-coupled network (Figure 1, Figure 2 and Figure 3) [4,5]. Especially at the borders of tracer-coupled networks, it is difficult to decide whether a cell displays a true tracer signal. Such manual decision can easily lead to over- or underestimation of the number of coupled cells. Furthermore, like the tracer neurobiotin, endogenous biotin is detected by avidin, causing elevated background levels (Figure 2) [21]. Astrocytes lacking tracer coupling are found in the LSO and further brain regions [4,5,11]. Furthermore, NG2 cells might be present in the LSO. Although they show panglial coupling with astrocytes in the corpus callosum [6], they were found in many brain regions to be neither tracer nor electrically coupled [8,11,26,27,28]. These predominantly uncoupled cells might express low amounts of biotin. In turn, cells might be assigned to the network just due to background labeling in previous analyses [4,5,11]. In this study, a semiautomated intensity-based cell detection method was used to overcome this problem. Only cells that exhibited an intensity 1.75-fold higher than the background were chosen as a part of the tracer-network.

### 3.2. Comparison of Approaches

In the following, the different approaches and their suitability to analyze various aspects of GJ coupling will be discussed. A simple and fast method is the manual “YX ratio” approach [4,5,10,11,15], but it lacks objectivity since the experimenter subjectively chooses the boundaries of the network. Furthermore, the number and density of the coupled cells have to be analyzed separately. An improvement of this strategy is provided by the automated “YX ratio” approach, where the basic idea remains the same but the ratio of the extensions is calculated automatically after determining cell positions. Here, the ratio is easy to calculate and more information about the tracer-coupled cells is immediately available. Furthermore, objectivity is achieved by automated identification of tracer-loaded cells. In the “Intensity + coordinates” approach, the somatic tracer signals are considered as well [14]. This provides an objective analysis strategy. However, elevated somatic signal intensities due to expression of endogenous biotin [21,22] can result in a distorted ratio. Another fast, intensity-based approach is “Intensity profiles” [7]. This approach is only partially objective as the chosen rectangles are placed manually. In addition, this method provides no information about number, location, and density of tracer-coupled cells. Furthermore, a sufficient difference in the emitted signal of the tracer-coupled network and the background intensity is required so that the Gaussian fit can be applied precisely.

Recently, a vector-based method was introduced. The “Vector sum” approach [12,13] is objective and denotes the preferential orientation of the tracer-coupled network, but only works in brain regions with defined borders and if the tracer-coupled network does not originate from the center, e.g., trigeminus [12,13], barrel Ctx [11], barreloid Th [7], and glomeruli of the olfactory bulb [29]. This approach is not applicable to tracer-coupled networks that are symmetric with respect to a point, e.g., in LSO [4], IC [5], HC [10,15], and Ctx [10,11]. The information regarding number and density of coupled cells is included, but a false positive/negative cell selection via automated image analysis might, e.g., result in an incorrect density of coupled cells.

To combine different aspects of analysis that were incompletely covered before—such as position and number of coupled cells as well as sufficient tracer-loading—we developed a new objective, vector-based method (“Vector means”) with subsequent meta-analysis. Here, the network is divided in four 90° sectors and the ratio of tracer extension is calculated in 15 steps via a rotating coordinate system. In combination with the semiautomated intensity-based cell detection method, this approach incorporates different information (shape, direction, number, and density of coupled cells) that allows a detailed characterization of the network topography and provides a fairly fast and automated analysis.

In an in silico model of artificially generated networks with predefined anisotropy, the automated “YX ratio” approach performed slightly better than the “Vector means” approach with subsequent meta-analysis. This was expected, because in an ideal environment, this approach will always result in the most accurate detection of anisotropy. However, the “Vector means” approach with subsequent meta-analysis offers comparable performance, while at the same time being less prone to errors caused by the experimenter. “YX ratio” relies on manually defining borders and orientation of the network extension, and thus only takes the four most distant tracer-coupled cells in the network into account. Our new, more automated approach is more robust and less sensitive to “outliers”, as it considers all tracer-coupled cells in the network.

### 3.3. Astrocyte Maturation and Network Topography

During maturation, astrocytes undergo a developmental transition from a nonpassive to passive state that is paralleled by reduction of *R*_M_ [17,18,24]. In contrast to the hippocampus, the size of LSO networks was independent from the maturation state of the tracer-loaded astrocyte [25]. However, we found a correlation of network topography and maturation state. nPA and PA gave preferentially rise to class 1 and class 2 networks, respectively (Table 1). This observation needs further confirmation in a developmental study, in which differently matured astrocytes are specifically targeted [17].

### 3.4. Tracer Coupling versus Electrical Coupling

Astrocytes were found to form isopotential networks throughout the central nervous system [30,31,32]. Remarkably, lack of tracer coupling does not per se translate into a lack of electrical coupling. In the barrel cortex, tracer coupling within or across septa is virtually absent [11]. However, electrical coupling, although weaker compared to electrical coupling within the barrels, persists [32]. Tracer-coupled networks in the LSO are predominantly anisotropic with a preferred orientation orthogonal to the tonotopic axis (this study and [4]). Nonetheless, LSO astrocytes are coupled to neighboring astrocytes and oligodendrocytes in any direction and accordingly will be electrically coupled to them. The heterogeneous tracer diffusion suggests that there is likely a heterogeneous electrical coupling as well—with a stronger electrical coupling orthogonal to than along the tonotopic axis. This so far unexplored feature must be addressed in future studies to better understand astrocyte functions in the LSO and how they might contribute to precise neuronal signaling.

### 3.5. Conclusions

Taken together, all ratio-based approaches displayed similar results regarding preferred network topography. However, they differ in the amount of information output. A high degree of objectivity is achieved by the semiautomated intensity-based cell detection method. Furthermore, the newly developed “Vector means” approach—together with our meta-analysis—exhibits a high capability to detect network anisotropy and provides detailed information about preferential orientation of tracer-coupled networks. Thus, our method will allow a reliable, fast, semiautomated, and objective analysis of tracer-coupled networks in future studies.

## 4. Materials and Methods

Experiments were performed on wild type C57Bl/6 mice of both genders in accordance with the German Animal Protection Law (TSchG) as well as guidelines for the welfare of laboratory animals released by the European Community Council Directive. In accordance with TSchG (Section 4, paragraph 3), no additional approval for postmortem removal of brain tissue was necessary. All chemicals were purchased from Sigma-Aldrich (St. Louis, MO, USA) or AppliChem (Darmstadt, Germany), if not stated otherwise.

### 4.1. Preparation of Acute Brainstem Slices

Acute coronal brainstem slices were prepared from animals at postnatal days 10 to 12, as described earlier [4,17]. After decapitation, brains were quickly transferred to an ice-cold solution containing (in mM) 26 NaHCO_3_, 1.25 NaH_2_PO_4_, 2.5 KCl, 1 MgCl_2_, 2 CaCl_2_, 260 d-glucose, 2 Na-pyruvate, and 3 myo-inositol, pH 7.4, bubbled with carbogen (95% O_2_, 5% CO_2_). 270-µm-thick slices were cut using a vibratome (VT1200 S, Leica; HM650V, Microtome, Microm International GmbH, Dreieich, Germany). To allow a priori identification of astrocytes slices were incubated for 30 min at +37 °C in 0.5–1 µM SR101 dissolved in artificial cerebrospinal fluid (ACSF) containing (in mM) 125 NaCl, 25 NaHCO_3_, 1.25 NaH_2_PO_4_, 2.5 KCl, 1 MgCl_2_, 2 CaCl_2_, 10 d-glucose, 2 Na-pyruvate, 3 myo-inositol, and 0.44 ascorbic acid, pH 7.4, bubbled with carbogen. Subsequently, slices were washed for 30 min at +37 °C in SR101-free ACSF. Thereafter, slices were kept at room temperature (RT).

### 4.2. Tracer Loading

Whole-cell patch-clamp experiments were performed at RT at an upright microscope equipped with infrared differential interference contrast (Eclipse FN1, Nikon, 60x water immersion objective, N.A. 1.0, Tokio, Japan) and an infrared video camera (XC-ST70CE, Hamamatsu, Shizuoka, Japan) using a double patch-clamp EPC10 amplifier and “PatchMaster” software (HEKA Elektronik, Lambrecht, Germany). The pipette solution contained (in mM) 140 K-gluconate, 5 EGTA (glycol-bis(2-aminoethylether)-*N*,*N*’,*N*’,*N*’-tetraacetic acid), 10 Hepes (N-(2-hydroxyethyl)piperazine-*N*’-2-ethanesulfonic acid), 1 MgCl_2_, 2 Na_2_ATP, and 0.3 Na_2_GTP, Ph 7.3. The pipette solution additionally contained a cocktail of the GJ-impermeable dye alexa fluor 568 (100 μM, Invitrogen) and the GJ-permeable tracer neurobiotin (1%, Vector Laboratories, Inc., Peterborough, UK) to mark the patched cell and label the coupling network, respectively [4,5,33]. Patch pipettes were pulled from borosilicate glass capillaries (GB150(F)28P, Science Products, Hofheim am Taunus, Germany) using a horizontal puller (P-87, Sutter Instruments, Novato, CA, USA) and had a resistance of 2–7 MΩ. Astrocytes were patched in the central part of the LSO, where the mediolateral and dorsoventral axes are roughly tangential and orthogonal to the tonotopic axis (Figure 1A). Astrocytes were patch-clamped and held −85 mV, which is close to their resting membrane potential [4,17]. Measurements were rejected if the series resistance exceeded 15 MΩ to ensure sufficient electrical and diffusional access to the patched cell [34]. The liquid junction potential was not corrected. SR101-positive cells were characterized by applying a standard step protocol ranging from −150 mV to +50 mV with 10 mV increments and step duration of 50 ms to determine their *I/V* relationship. The resulting current traces were sampled at 50 kHz and online filtered at 2.9 kHz. Data were analyzed using “IGOR Pro” Software (WaveMetrics, Lake Oswego, OR, USA). After calculating the linear regression curve, two types of astrocytes could be distinguished according to their respective regression coefficient (see [18]): (1) nPA (*R*^2^ < 0.9983) and (2) PA (*R*^2^ ≥ 0.9983). After 30 min of tracer and dye loading, the patch pipette was carefully withdrawn and the slice was immediately fixed overnight (about 20 h) in 4% paraformaldehyde (PFA) at +4 °C.

### 4.3. Visualization of Coupled Cells

Gap junctional networks were visualized as described earlier [4,5,33]. Neurobiotin was identified using avidin AF 488 (50 μg/mL, Invitrogen, Carlsbad, CA, USA). Fixed slices were further processed at RT. Slices were washed three times in phosphate buffered solution (PBS, containing NaCl, Na_2_HPO_4_·2 H_2_O, NaH_2_PO_4_·H_2_O; pH 7.4). Membrane permeabilization was achieved by incubation in 0.25% triton X-100 for 30 min. Thereafter, slices were washed again in PBS. Neurobiotin was identified incubating slices for 3 h with avidin alexa fluor 488 (50 μg/mL, Invitrogen) and slices were washed again.

To determine the position of the tracer-coupled networks within the LSO, we subsequently processed the tissue immunohistochemically for GlyT2. These transporters are mainly localized in presynaptic terminals that contact principal cells throughout the LSO and thereby mark the nucleus [35]. As described earlier [4,36], avidin-labeled slices were again permeabilized for 30 min in 0.25% triton X-100. Unspecific binding sites were blocked for 1 h in a solution containing 2% bovine serum albumin (BSA), 11.1% normal goat serum (NGS; PAA laboratories, Cölbe, Germany), and 0.3% triton X-100. The slices were then incubated overnight (about 20 h) at +4 °C with primary antibody (rabbit anti-GlyT2, AB1773, Millipore, Burlington, MA, USA) diluted 1:10,000 in 1% BSA, 1% NGS, and 0.3% triton X-100. The next steps were again performed at RT. After washing in PBS, slices were incubated for 90 min with the secondary antibody (goat anti-rabbit alexa fluor 647, A-27040, Invitrogen) diluted 1:300 in 1% BSA, 1% NGS, and 0.3% triton X-100. Finally, slices were washed in PBS and mounted in 2.5% Dabco on glass slides.

### 4.4. Confocal Microscopy

SR101-labeling, network tracing, and immunohistochemical labeling were documented with a confocal microscope—Zeiss LSM700 (EC Plan-Neofluar 10×/0.3)—in combination with ZEN software (Zeiss, Oberkochen, Germany). Fluorophores were detected as described before [4]. To improve the quality of confocal micrographs and reduce background fluorescence, we used a Kalman filter (averaging of four identical image sections). In all experiments, a single optical plane was documented. 

### 4.5. Reconstruction of Gap Junction Networks

Confocal images were processed using Fiji software [37]. Since not only the tracer-coupled network but also cells that express endogenously biotin promote avidin signals, a semiautomated intensity-based cell detection method was applied to decide objectively which of these cells part of the tracer-loaded network were. At first, all identified cells were marked as a region of interest (ROI) with the oval brush selection tool (gray circles and ellipses in Figure 2A). The ROI manager was then used to measure the mean gray values and the x- and y-coordinates. Then, three of these cells, which were distant from the patched cell but still inside of the LSO borders, were chosen (white squares in Figure 2A,C). The mean gray values of these three ROIs correspond to the background intensity. For subsequent analysis of the tracer-coupled networks, cells that exhibited a signal with intensity 1.75 times the intensity background level were chosen.

### 4.6. Analysis of Network Topography

Patch-clamped astrocytes, which were initially filled with tracer, were identified via dialysis of their soma with alexa fluor 568. Tracer-coupled cells were visualized via avidin alexa fluor 488 (see Section 4.3). In 5/6 approaches to analyze the topography of tracer-labeled network two values were obtained, which resemble extent of the networks in two directions orthogonal to each other. Tracer-labeled networks were assigned to three classes depending on the ratio *R*, defined as the quotient of extension (Figure 3A–D,F): (1) *R* > 1.1, oval-shaped orthogonally to the tonotopic axis, (2) 0.91 (1/1.1) < *R* ≤ 1.1, spheroidal-shaped, and (3) *R* ≤ 0.91 (1/1.1), oval-shaped along the tonotopic axis [4,5]. In order to analyze whether there is a preferred tracer-labeled network shape and orientation, we analyzed the normalized extension orthogonal to versus along the tonotopic axis. Data were normalized to values of extension along the tonotopic axis.

#### 4.6.1. Manual “YX Ratio”

The most often used approach for the analysis of network anisotropy is to calculate the ratio *R* by dividing the extension of two orthogonal directions *y* and *x* (Equation (1); Figure 3A_1_) [4,5,10,11,15]:

(1) $$R=\frac{y}{x}$$

where *R* is the ratio of the two axes, *y* is the tangent of the tonotopic axis, and *x* the orthogonal of that tangent. 

#### 4.6.2. Automated “YX Ratio”

After applying the cell detection method (see Section 4.5), the “YX ratio” can be quantified automatically from the ROIs instead of manually measuring the two extensions of the tracer-coupled network. Here, the maximal values for the positive and the minimal values for the negative coordinates for each direction were determined to calculate the lengths *x* and *y*. As this is just the automation of the manual “YX ratio” approach, their equation is the same (Equation (1); Figure 3B_1_).

#### 4.6.3. “Intensity + Coordinates”

The ratio *R* can also be calculated via the intensities and the coordinates of the ROIs by dividing the sum product of the intensity and the *y*-coordinate of the ROIs by the sum product of the intensity and the *x*-coordinate (Equation (2); Figure 3C_1_) [14]:

(2) $$R=\frac{\sum_{1}^{i}\left|I_{i}y_{i}\right|}{\sum_{1}^{i}\left|I_{i}x_{i}\right|}$$

where *I_i_* is the mean gray value of each ROI and *y_i_* and *x_i_* are the coordinates of the respective centroid.

#### 4.6.4. “Intensity Profiles”

In an alternative approach, two rectangles orthogonal to each other with a width of 100 µm were chosen and their signal intensity profiles were measured (Figure 3D_1_; [7]). Then, the FWHMs of the two graphs were calculated by fitting the data to a Gaussian curve. The ratio *R* is given by the quotient of theses FWHMs (Equation (3)):

(3) $$R=\frac{FWHM_{y}}{FWHM_{x}}$$

where *FWHM_y_* is the full-width at half-maximum of the intensity profile in *y*-direction and *FWHM_x_* is the full-width at half-maximum of the intensity profile in *x*-direction. However, the cell detection method could not be applied here.

#### 4.6.5. “Vector Sum”

A vector-based approach was also used to describe astrocyte network anisotropy [12,13]. Here, the angle between the vector of preferential orientation of the tracer-coupled network and a predefined direction, here the *y*-axis, was calculated (Equation (4); Figure 3E_1_):

(4) $$\alpha \;=\cos^{-1}\left(\frac{\sqrt{{\left(y_{s}-y_{0}\right)}^{2}}}{\sqrt{{\left(x_{s}-x_{0}\right)}^{2}+{\left(y_{s}-y_{0}\right)}^{2}}}\right)$$

where *α* is the angular difference in degrees, (*x*_s_, *y*_s_) are the coordinates of the sum vector, and (*x*_0_, *y*_0_) is the position of the patched cell.

#### 4.6.6. “Vector Means”

In order to achieve an objective, automated calculation that covers all analysis aspects in terms of anisotropy, preferred orientation, and cell distribution, we developed a new vector-based method with subsequent meta-analysis (see Section 4.6.8.). First, the cell detection method was applied to determine which of the cells belong to the tracer-coupled network. The network was then divided into four 90° sectors and the sum vector for each sector is calculated (Figure 3F_1_). The length was normalized to the number of cells in each sector. The ratio *R* is the quotient of the normalized *y* value and the normalized *x* value (Equation (5)):

(5) $$R=\frac{\frac{\left|\vec{y_{1A}}\right|}{n_{1A}}+\frac{\left|\vec{y_{1B}}\right|}{n_{1B}}}{\frac{\left|\vec{x_{2A}}\right|}{n_{2A}}+\frac{\left|\vec{x_{2B}}\right|}{n_{2B}}}$$

where $\left|\vec{y_{1A}}\right|$, $\left|\vec{y_{1B}}\right|$, $\left|\vec{x_{2A}}\right|$, $\left|\vec{x_{2B}}\right|$ are the absolute values of the sum vectors of the sectors 1A, 1B, 2A, and 2B, respectively, and $n_{1A}$, $n_{1B}$, $n_{2A}$, and $n_{2B}$ are the number of cells in respective sectors.

#### 4.6.7. Meta-Analysis

The preferred orientation of the tracer-coupled networks was calculated by adding a meta-analysis to our vector-based method (see Section 4.6.6). Here, the coordinate system was rotated and the ratio was recalculated in steps of 15 (Figure 4). Then, a sinusoidal function (Equation (6)) was fitted to the data:

(6) $$R=A_{0}+A\sin\left(\omega \alpha +\left(\varphi +\frac{3}{4}\pi \right)\right)$$

where *A_0_* is the offset, *ω* is the circular frequency, *α* is the angle, and *ϕ* is the phase shift.

The highest Ratio (*R_max_ = A_0_ + A*) of the fit gives the angle of maximal anisotropy of a single network. The networks are therefore affiliated to being oval- (*R_max_* > 1.1, wave symbol) or round-shaped (*R_max_* ≤ 1.1, line). A Gaussian fit (Figure 4D_1_) was then used to determine the preferential orientation of all networks.

We designed an Excel document, which allows the user to analyze easily GJ networks. This includes the different approaches as well as the meta-analysis described in Section 4.6 that are depicted in Figure 3 and Figure 4, respectively (see Supplementary Material).

#### 4.6.8. Generation of Artificial Networks In Silico

Using MATLAB (R2016B, MathWorks, Natick, MA, USA), cells were first placed randomly into a rectangular space, setting the network center separately as the starting point of tracer diffusion (patched cell). The minimal distance between each cell, including the center, was 15 µm. An elliptic border was defined with *R* ranging from 1.00 up to 1.20 in 0.05 steps and all cells outside of it were discarded. As the tracer signal declined with increasing distance to the patched cell, the relative brightness of the cells was calculated using exponential decay from the center with λ = 80 µm along the long axis of the ellipse. For each cell, an individual λ was calculated by scaling it linear with the following ratio: distance from the network center to the border of the ellipse along the axis of the given cell divided by the distance from the network center to the border of the ellipse along the long axis. Resembling the 1.75-fold background used in our experiments, a threshold of 0.2 was used for in silico networks. Therefore, cells of low brightness were discarded resulting in networks of different anisotropy containing between 60 and 80 cells.

### 4.7. Statistics

Data are provided as mean ± SEM. Data were statistically tested using WinSTAT (R. Fitch Software, Bad Krozingen, Germany). Differences in distribution of classes between nPA and PA were tested with Χ^2^ test. To analyze, whether nPA- and PA-derived networks exhibited the same size we first proofed that both population exhibited normal distribution using Kolmogorov–Smirnov test. As data were normally distributed we subsequently utilized a standard two-sided Student’s *t*-test. *p* represents the error probability. *n* represents the number of cells or experiments/animals.

## Figures and Tables

**Table 1:** Network topography correlates with astrocytic basic membrane properties.

Study	Approach	nPA (classes)			PA (classes)			*p*
		1	2	3	1	2	3	
This study (LSO)	YX ratio (manual)	79%	14%	7%	40%	50%	10%	1.6096 × 10^−14^
	YX ratio (automatic)	64%	36%	0%	50%	30%	20%	3.4229 × 10^−6^
	Intensity + coordinates	93%	0%	7%	60%	20%	20%	9.0177 × 10^−11^
	Intensity profiles	57%	43%	0%	30%	70%	0%	3.1601 × 10^−9^
	Vector means	79%	21%	0%	30%	60%	10%	2.3325 × 10^−25^
Previous studies								
[4] (LSO)	YX ratio (manual)	58%	33%	8%	39%	39%	22%	6.7850 × 10^−5^
[5] (IC)	YX ratio (manual)	71%	21%	7%	43%	43%	13%	1.0375 × 10^−7^

Differences in the distribution were statistically analyzed using a Χ^2^ test. nPA: nonpassive astrocyte; PA: passive astrocyte; class 1: oval, orthogonal to tonotopic axis; class 2: spherical; class 3: oval, along to tonotopic axis (see Section 4.6.).

## Supplementary Materials

Supplementary materials can be found at https://www.mdpi.com/1422-0067/20/11/2821/s1.

## Abbreviations

ACSF	Artificial cerebrospinal fluid
BSA	Bovine serum albumin
Ctx	Cortex
Cx	Connexin
FWHM	Full-width at half-maximum
GJ	Gap junction
HC	Hippocampus
IC	Inferior colliculus
LSO	Lateral superior olive
Nb^+^	Neurobiotin-positive
NGS	Normal goat serum
nPA	Nonpassive astrocyte
PA	Passive astrocyte
PBS	Phosphate buffered solution
PFA	Paraformaldehyde
*R*	Ratio
*R* ^2^	Regression coefficient
RT	Room temperature
ROI	Region of interest
SR101	Sulforhodamine 101
Th	Thalamus
