# Supplementary material for: A Vector-Based Method to Analyze the Topography of Glial Networks
Source: Int J Mol Sci. 2019 Jun 10;20(11):2821. doi: 10.3390/ijms20112821 (PMC6600595; doi:10.3390/ijms20112821)
Supplement: Supplementary file 1 [file ijms-20-02821-s001.zip › ijms-516213-supplementary/ijms-516213 suppl.pdf]

International Journal of *Molecular Sciences*

Supplementary Table 1. Distribution of classes.

Approach	Oval (orthogonal to tonotopic axis)	Spherical	Oval (along to tonotopic axis)
YX ratio (manual)	63%	29%	8%
YX ratio (automatic)	54%	33%	13%
Intensity + coordinates	79%	8%	13%
Intensity profiles	46%	54%	0%
Vector means	58%	38%	4%

The “Vector sum” approach is omitted here, as its results are not based on ratios. n = 24 slices/19 animals.
